# Tremor Suppression by Rhythmic Transcranial Current Stimulation

**DOI:** 10.1016/j.cub.2013.01.068

**Published:** 2013-03-04

**Authors:** John-Stuart Brittain, Penny Probert-Smith, Tipu Z. Aziz, Peter Brown

**Affiliations:** 1 Nuffield Department of Clinical Neurosciences, University of Oxford, Oxford OX3 9DU, UK; 2 Nuffield Department of Surgical Sciences, University of Oxford, Oxford OX3 9DU, UK; 3 Department of Engineering, University of Oxford, Oxford OX1 3PJ, UK

## Abstract

Tremor can dominate Parkinson’s disease and yet responds less well to dopaminergic medications than do other cardinal symptoms of this condition [1, 2]. Deep brain stimulation can provide striking tremor relief, but the introduction of stimulating electrodes deep in the substance of the brain carries significant risks, including those of hemorrhage [3]. Here, we pioneer an alternative approach in which we noninvasively apply transcranial alternating current stimulation (TACS) over the motor cortex [4, 5] to induce phase cancellation of the rest tremor rhythm. We first identify the timing of cortical oscillations responsible for rest tremor in the periphery by delivering tremor-frequency stimulation over motor cortex but do not couple this stimulation to the on-going tremor—instead, the rhythms simply “drift” in and out of phase alignment with one another. Slow alternating periods of phase cancellation and reinforcement result, informing on the phase alignments that induce the greatest change in tremor amplitude. Next, we deliver stimulation at these specified phase alignments to demonstrate controlled suppression of the on-going tremor. With this technique we can achieve almost 50% average reduction in resting tremor amplitude and in so doing form the basis of a closed-loop tremor-suppression therapy that could be extended to other oscillopathies.

## Graphical abstract

**Graphical Abstract:**

**Figure:**
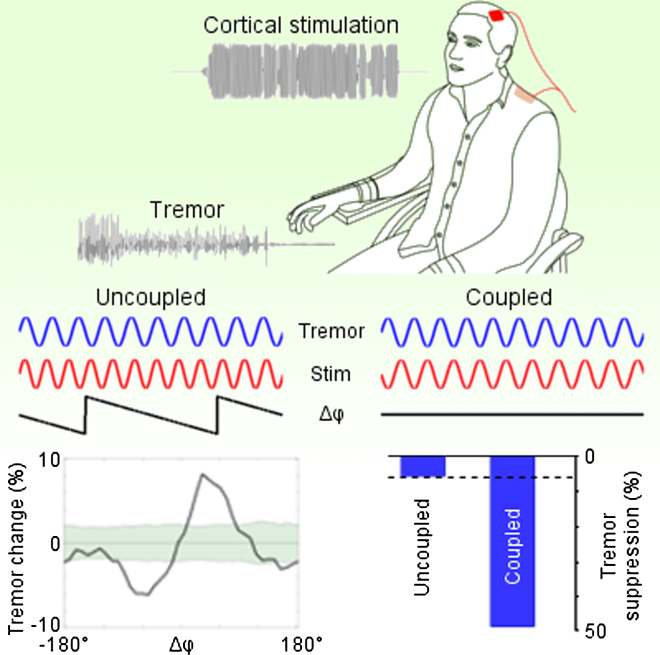

**Highlights:**

► Phase-cancelling cortical stimulation attenuates Parkinsonian tremor ► Prolonged stimulation may invoke adaptive mechanisms ► Phase cancellation may provide a generic treatment approach to oscillopathies ► TACS provides a convenient probe of cortical circuit dynamics in humans

## Results

### Experiment 1: Phase Drift

**Figure 1:**
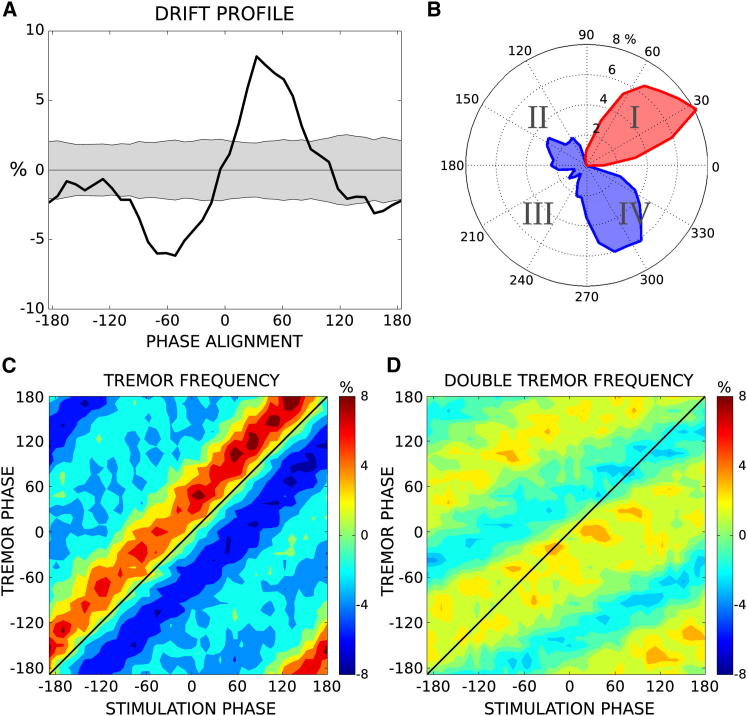
Exemplar Responses in Patient 11 (A) Mean percentage change in tremor amplitude displayed relative to phase alignment between the tremor and TACS signals. (B) Polar representation where red indicates periods of tremor excitation and blue periods of tremor suppression. (C) Absolute phase drift-response during tremor-frequency stimulation. (D) Absolute phase drift-response during first-harmonic stimulation. See also Figure S1.

Drift results from a single patient displaying tremor-dominant Parkinson’s disease are presented in Figure 1 (see Supplemental Experimental Procedures available online). Tremor amplitude is clearly dependent on the phase alignment between TACS and tremor signals (Figure 1A). Tremor amplitudes in this case are demonstrably lower in quadrant IV (6.2% decrease; see Figure 1B) and higher when tremor leads TACS in quadrant I (8.2% increase). Tremor amplitude can also be assessed in relation to all combinations of stimulation and tremor phase (Figure 1C). In the case of tremor-frequency stimulation, we confirm a diagonal relationship, i.e., tremor amplitude depends only on the phase difference and not the absolute phase of tremor and stimulation waveforms.

Tremor syndromes are often associated with cortical activity at both tremor frequency and at double this frequency [6]. We therefore tested whether transcranial stimulation at the basic tremor rhythm or its first harmonic was more effective in modulating peripheral tremor. Maximum attained suppression and excitation amplitudes were both clearly lower in the harmonic case than during tremor-frequency stimulation (Figure 1D).

**Figure 2:**
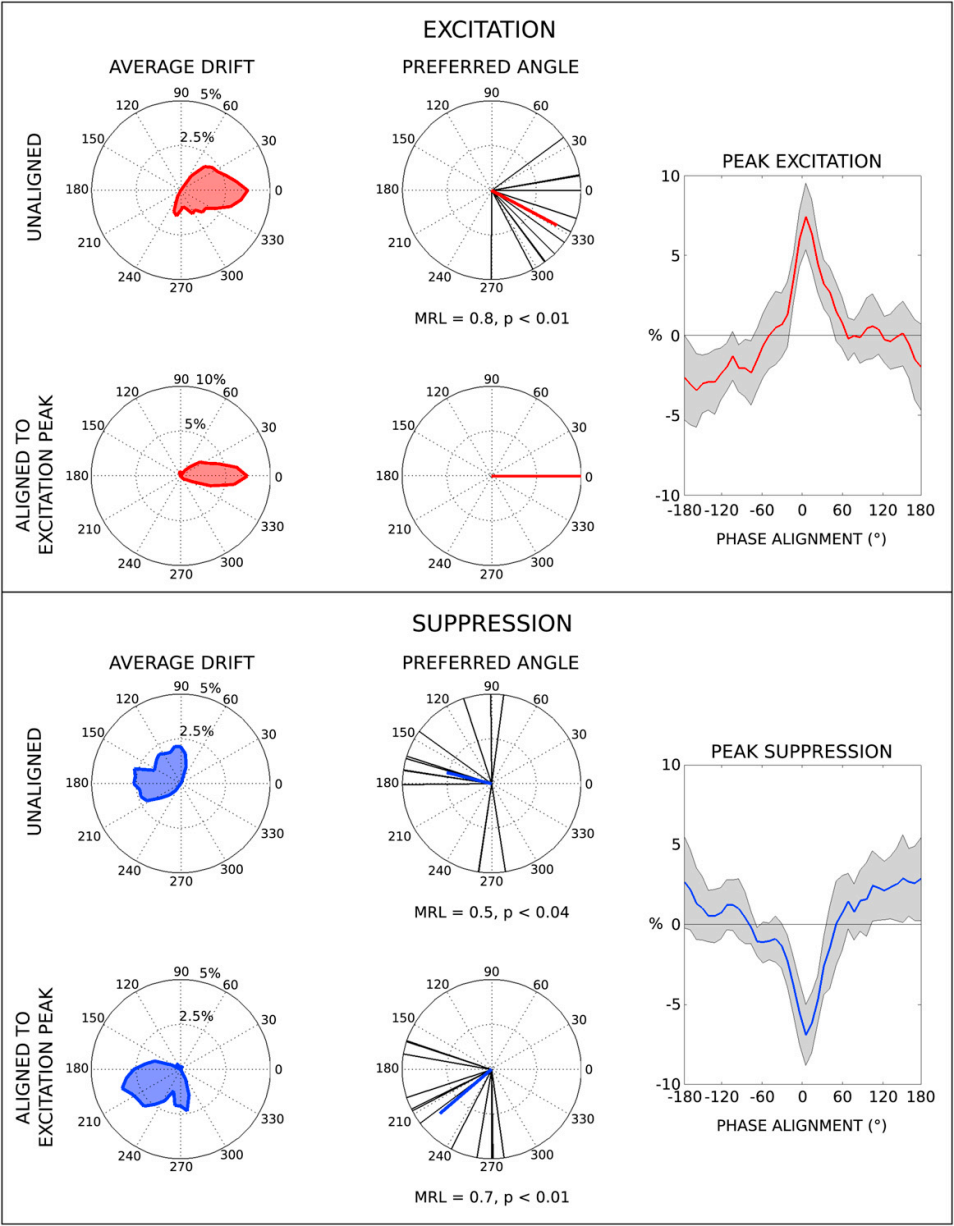
Group Data from the Drift Experiment Top: Excitation response. Drift profiles averaged across patients are presented alongside their individual preferred orientations (black lines) and mean orientation vector (red line). Similar profiles are displayed after realignment to the mean orientation angle, displayed under their respective counterparts. Tremor amplitude is also presented aligned to individual peak excitation angles. Bottom: Suppression response (as top panel). See also Figure S2.

Drift results from a cohort of n = 12 patients displaying tremor-dominant Parkinson’s disease are presented in Figure 2. Stimulation induced a small fluctuation in tremor frequency away from the patients’ intrinsic tremor frequency, causing a mean absolute change (±SD) of 0.4 ± 0.7 Hz relative to the sham condition (see Figure S1). Because of this and the difference in intrinsic tremor frequency, the drift interval (time to complete a full cycle of phase alignment) varied between patients, taking on average 3.3 ± 2.2 s. When aligned to individual peak suppression angles, tremor amplitude decreased by an average of 6.9% ± 3.4% (t(11) = −7.06, p < 0.001, left-tailed test, corrected for two multiple comparisons by the false discovery rate [FDR], see Supplemental Experimental Procedures). Conversely, alignment to the peak excitation angle saw tremor increase by 7.4% ± 3.7% (t(11) = 7.06, p < 0.001, right-tailed test).

The phase alignments producing the greatest increase and decrease in tremor amplitude were then determined per patient. Group average drift profiles and preferential phase angles are presented in Figure 2. Preferential phase alignment can be considered at the group level by calculating the mean resultant length (MRL) and comparing this value to an empirical null distribution (see Supplemental Experimental Procedures). The phase alignment for excitation displays a significant group preference toward quadrant IV (MRL = 0.8, p < 0.01) with a mean orientation of −28° which, given a 5 Hz tremor, is equivalent to a temporal delay of 16 ms with TACS leading tremor. The orientation preference for peak suppression was likewise significant (MRL = 0.5, p < 0.04) with a spread of preferred orientations spanning quadrants II and III.

The fact that peak suppression and excitation angles were not in antiphase raises the possibility that either the underlying cortical activity is not sinusoidal and symmetrical at any point in time or that we are modulating independent cortical rhythms. To clarify this, we rotated all vectors relative to their individual excitation peaks. When aligned in this way, the peak suppression angle shows a significant preference toward quadrant III with a mean orientation of −139° (MRL = 0.8, p < 0.01). A weak, often asymmetric, entrainment is also evident in some patients (Figures S2A and S2B). Thus, peak suppression and excitation are themselves phase locked in line with interaction with a single cortical rhythm, albeit one that may not be perfectly sinusoidal and may therefore have harmonic elements.

Our cohort also confirmed that stimulation at tremor frequency was more effective at suppressing tremor than stimulation at the first-harmonic rhythm (mean change −7.1% ± 3.6% versus −4.0% ± 3.6%, paired t(8) = −3.26, p < 0.02). Likewise, the relative increase in tremor amplitude was significantly greater during tremor-frequency stimulation than during first-harmonic stimulation (7.4% ± 3.8% versus 4.3% ± 2.8%, paired t(8) = 3.02, p < 0.02). Finally, weaker rhythms appear more amenable to intervention (Figure S2C).

### Experiment 2: Phase Tracking

Experiment 1 provided proof of the principle that phase-aligned rhythmic stimulation can attenuate Parkinsonian tremor through phase cancellation, but the suppressive effects were modest and mixed with periods during which tremor was exaggerated. We reasoned that sustained phase cancellation would lead to greater suppression of peripheral tremor through adaptation. To achieve this, we again stimulated at tremor frequency in a subset of five patients, but this time tracked the phase of the peripheral tremor so as to continuously deliver stimulation at a specified phase alignment with respect to the on-going tremor (and hence the cortical oscillations).

**Figure 3:**
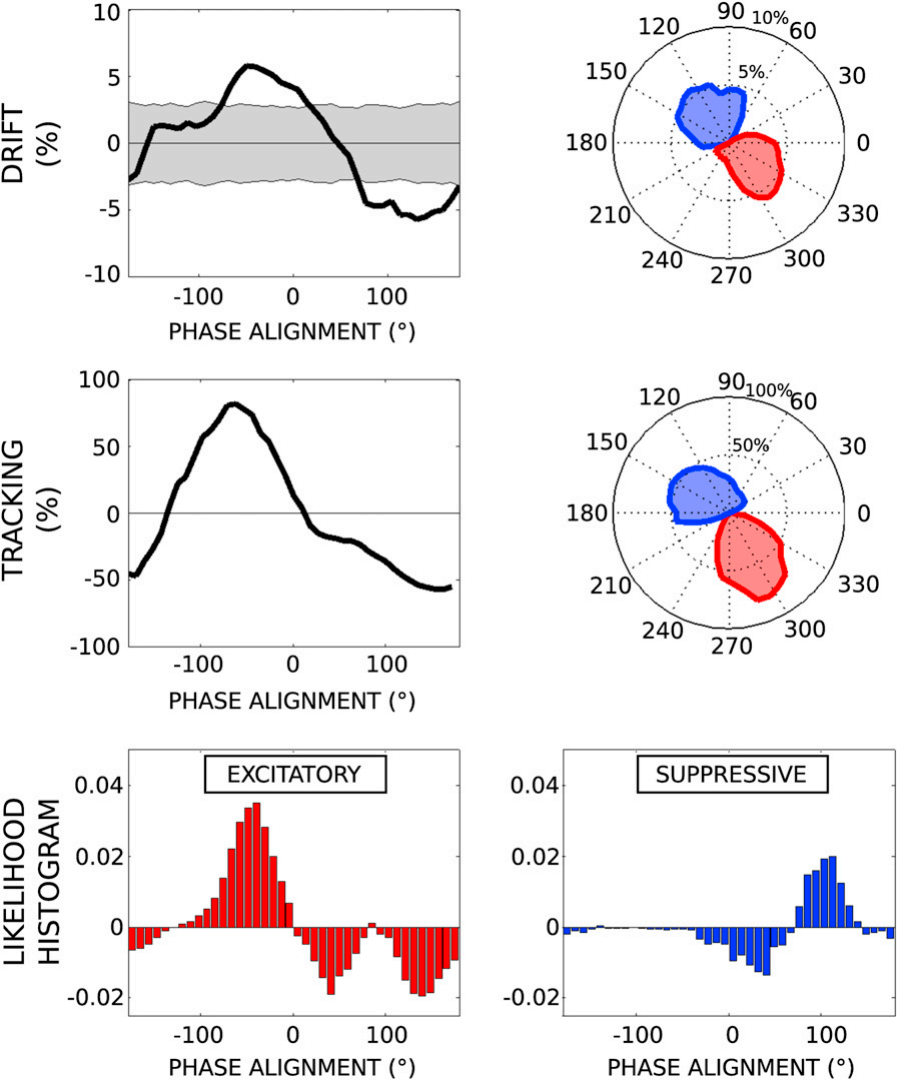
Phase Tracking Increases Effect Size Top: Drift response. Tremor amplitude versus phase alignment for case 10 from experiment 1. Middle: Phase tracking. Tremor amplitude versus phase alignment under sustained phase alignment (30 s segments over 9° increments). Note the order of magnitude change in scale relative to the drift analysis. Bottom: Normalized phase-difference histograms for excitatory and suppressive segments. A clear peak is visible in both cases at the expected excitatory/suppressive phase angle. See also Figure S3.

We first sought to reconstruct the drift profile observed during experiment 1 to confirm its utility in informing on effective phase alignments. Thus in case 10 we delivered phase-locked stimulation for 30 s at 9° increments (random ordering) around the phase circle (40 segments). We compute the mean tremor amplitude per segment and display this versus orientation angle (Figure 3). Compared to the drift response, the two profiles are remarkably similar (correlation coefficient R^2^ = 0.75; cross-correlation peaks at zero lag). However, one clear distinction between the drift and tracking results was an order of magnitude increase (×13.9 best fit) in effect size under tracking.

**Figure 4:**
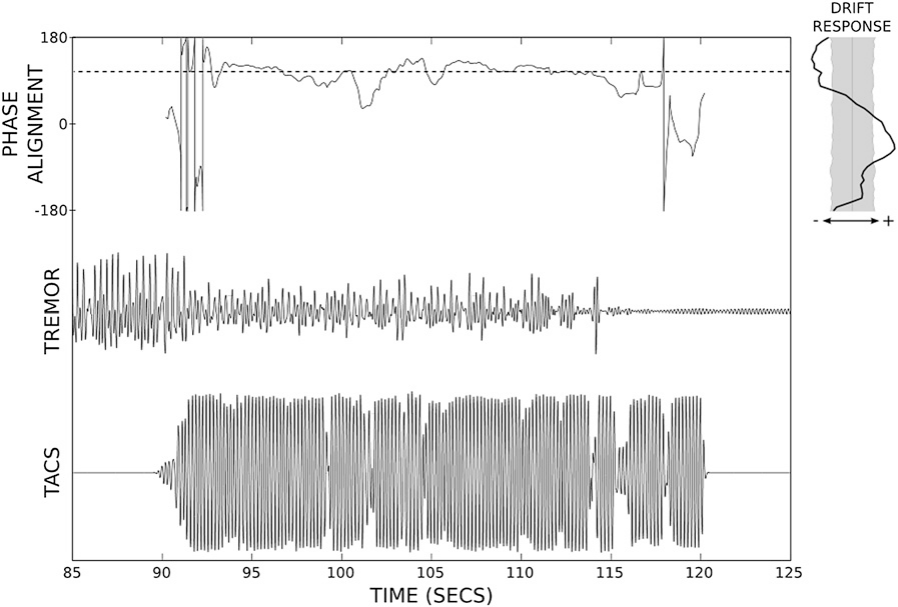
Phase Tracking Can Lead to Complete Tremor Suppression Stimulation and tremor signals for case 10 are displayed alongside their phase alignment. The patient’s drift response is also presented for comparison.

To confirm that our results related specifically to the phase of stimulation, we assessed the phase difference between peripheral tremor and stimulation during blocks of observed tremor suppression and excitation (see Supplemental Experimental Procedures). Phase-difference histograms demonstrate that during tremor suppression (similarly excitation), phase alignment favored those angles predicted through the drift (and tracking) analysis. Overall, suppression was associated with a mean tremor reduction of 52% (a factor of 2.08) and an increase of 348% (a factor of 3.48). A sample trace showing suppressive phase-locked stimulation interacting with a 30 s tremor segment is presented in Figure 4. Note how rest tremor is completely suppressed before and beyond the end of the stimulation train.

Phase-locked stimulation was trialed on a further four patients (cases 6, 8, 13, 14). Across all five patients, 30 s of sustained stimulation about the peak suppression angle led to a reduction in tremor amplitude of between 21% and 53% (mean 42% ± 13%, representing a factor decrease of 1.80 ± 0.35).

## Discussion

We have demonstrated a potentially powerful noninvasive therapy for resting tremor in Parkinson’s disease (PD). TACS provides both a means to probe the tremor circuit, determining the optimal parameters for tremor suppression, and the rhythmic stimulation necessary for phase cancellation.

Rhythmic transcranial stimulation has already demonstrated behavioral influences on memory [7] and motor circuits [4, 5] in healthy subjects. It is believed that TACS induces subthreshold changes in the membrane potentials of local neurons that then shape the likelihood of affected neurons firing in response to natural inputs [8]. Thus the effects of TACS are dependent on spontaneous activity. This has been confirmed experimentally, where the strength of entrainment of cortical spiking induced by stimulation has been shown to be dependent upon the behavioral state of the animal [9]. TACS therefore exerts a modulatory but nondominant influence.

Our approach utilizes the peripheral tremor as a proxy for cortical oscillatory activity, providing a noninvasive means of identifying phase dependency for cortical phase cancellation. Stimulation was significantly more effective when delivered at tremor frequency than at the first harmonic rhythm. This is of particular interest because a growing body of evidence suggests, at least in PD, that the fundamental and first harmonic rhythms play distinct roles in tremor generation [10]. Indeed, it is believed that these rhythms might originate from separate cortical sources [11] and have been attributed with distinct functional roles [12]. The extent of sensory contributions to the central tremor-frequency oscillation is also debated [10, 13] and whether we are directly modulating efferent drive or suppressing inputs to the cortex such as afferent reinforcement remains to be clarified.

Prolonged phase-locked stimulation invoked much stronger amplitude effects than uncoupled stimulation. This supports the premise that TACS may induce adaptive changes in the underlying tremor circuit (see [8]). Finally, it should be highlighted that interference through phase cancellation preferentially targets tremor-related oscillations (those phase locked to the tremor) and should therefore leave physiological patterns of synchronization relatively unaffected (see Figure S3), even when those frequencies overlap.

Tremor reduction when the optimal suppressive phase was sustained for 30 s was almost 50% across our Parkinson’s disease patients. This is less than the effect generally achieved with deep brain stimulation but the latter is associated with significant risks and is not suitable for all patients. Transcranial phase cancellation, on the other hand, is noninvasive and the effects achieved even in this pilot study were not trivial; by way of comparison, the tremor suppression was equivalent to the efficacy of Primodone and Propranolol, two established and widely used treatments for essential tremor [14]. Indeed, it is possible that the efficacy of phase-cancelling transcranial stimulation could be further improved by a higher resolution of frequency tracking, given that stimulation during tracking only matched tremor frequency to the nearest Hertz and in and of itself induced changes in the tremor frequency. Longer periods of stimulation at the optimal phase and frequency might also improve efficacy.

In conclusion, we have demonstrated attenuation of Parkinsonian rest tremor by noninvasive phase cancellation. Although the approach remains to be refined and sustained benefit with chronic stimulation demonstrated, it is exciting in that it may potentially leverage intrinsic mechanisms of plasticity and leave oscillations subserving physiological processes relatively unaffected. In the future, phase-cancelling stimulation could be delivered chronically with minimally invasive subcutaneous or extradural electrodes. In this form, phase cancellation could provide a generic approach to therapeutic intervention, suitable not only for tremor but also for other disease impairments caused by synchronized oscillations [15, 16].

## Experimental Procedures

The study was performed with the approval of the NRES Ethics Committee South Central-Oxford C and the informed, written consent of all subjects. See Supplemental Experimental Procedures for further details.

## Supplemental Information

Document S1. Supplemental Experimental Procedures, Figures S1–S3, and Table S1
