# Supplementary material for: Tremor Suppression by Rhythmic Transcranial Current Stimulation
Source: Curr Biol. 2013 Mar 4;23(5):436–40. doi: 10.1016/j.cub.2013.01.068 (PMC3629558; doi:10.1016/j.cub.2013.01.068)
Supplement: Document S1. Supplemental Experimental Procedures, Figures S1–S3, and Table S1 [file mmc1.pdf]

**Current Biology, Volume *23***

# Supplemental Information

# Tremor Suppression by Rhythmic Transcranial Current Stimulation

**John-Stuart Brittain, Penny Probert-Smith, Tipu Z. Aziz, and Peter Brown**

## Inventory of Supplemental Information

**Figure S1** provides an illustrative example of the frequency-shift phenomenon mentioned in the main text. This diagram serves not only to provide evidence of this shift, but also characterises the phenomenon (i.e. a single deflection that quickly settles into a new homeostatic rhythm). Figure S1 is referenced (and references) Figure 1, and is referred to in the main text when discussing the frequency shift-phenomenon.

**Figure S2** provides additional requested material relating to phase-entrainment and amplitude effects. Panel A provides case-by-case likelihood plots for tremor vs stimulation phase-alignment, along with their respective phase-synchronisation indices (PSI). This panel demonstrates graphically the weak entrainment effect of stimulation on tremor, and provides additional characterising information, such as asymmetries in the response. The information in panel A leads directly to consideration of the PSI as predictive of stimulation efficacy, as illustrated through the regression plot of panel B. Panel C provides a measure of stimulation efficacy versus tremor amplitude, indicating that low-amplitude tremors are more likely to respond to stimulation. Figure S2 is referenced (and references) Figure 2, which both present group statistics related to experiment 1. The discussion of phase-entrainment especially has been extended with reference to Figure S2 in the main text.

**Figure S3** provides initial evidence that physiological processing, as characterised by performance on a manual dexterity task, remains unimpaired in a subsample of patients. Figure S3 is referenced (and references) Figure 3, which both relate to results obtained through phase-tracking stimulation (experiment 2). The issue of physiological processing is discussed in the main text, with reference to Figure S3.

**Table S1** provides more detailed patient information and is referenced in supplemental experimental procedures.

**Supplemental experimental procedures** have been introduced which significantly expand on and clarify the experimental procedures from the previous manuscript iteration.

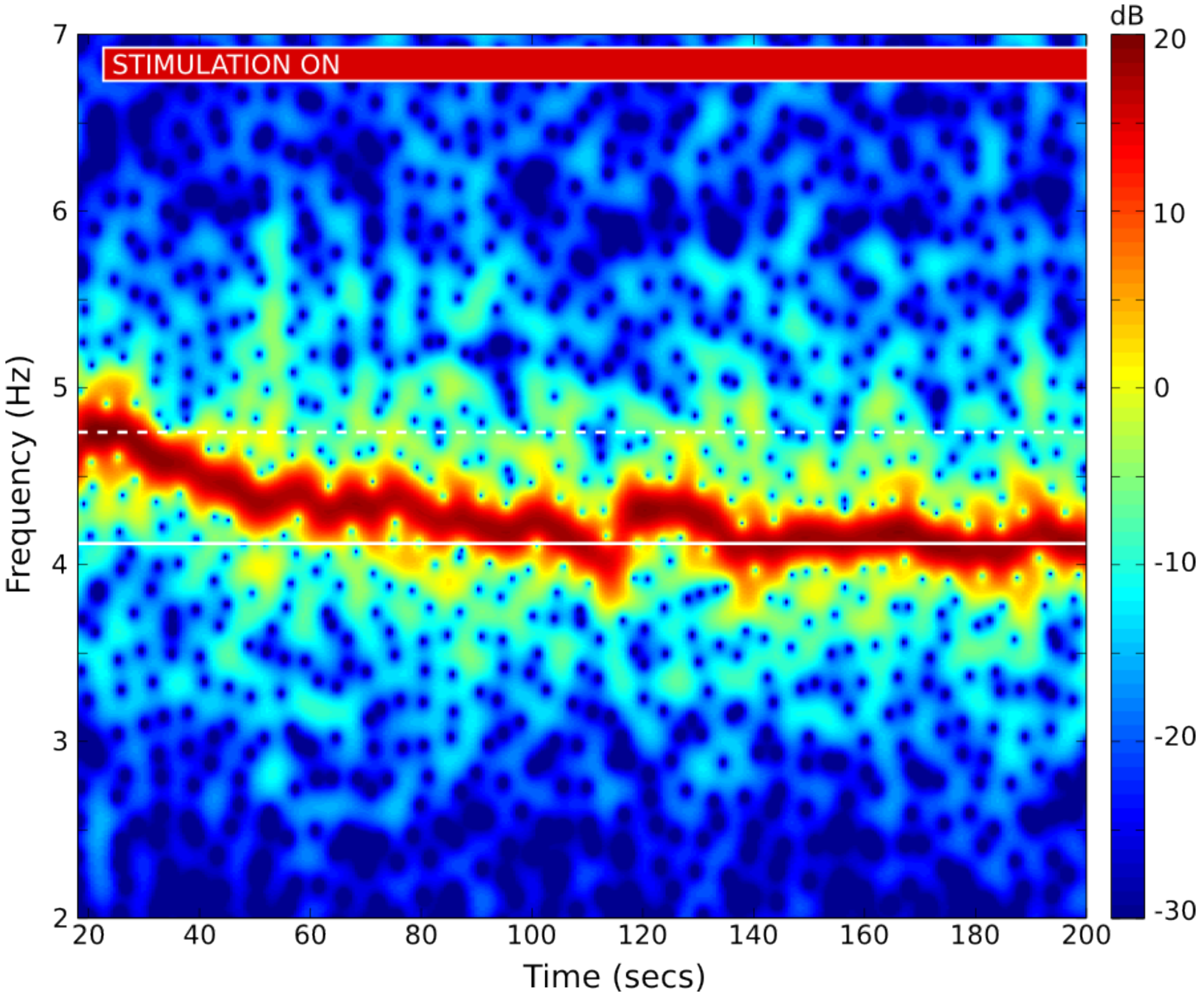


**Figure S1 (related to Figure.1).** Under tremor-frequency stimulation the frequency of the peripheral resting tremor self-modified and settled on a new frequency in 9 of our 12 patients from Experiment 1. This frequency remained fixed for the duration of the stimulation session. Diagram shows a sample spectrogram of the primary accelerometer channel as stimulation is imposed. Note the change in frequency from 4.8 Hz (dashed white line) to 4.2 Hz (solid white line).

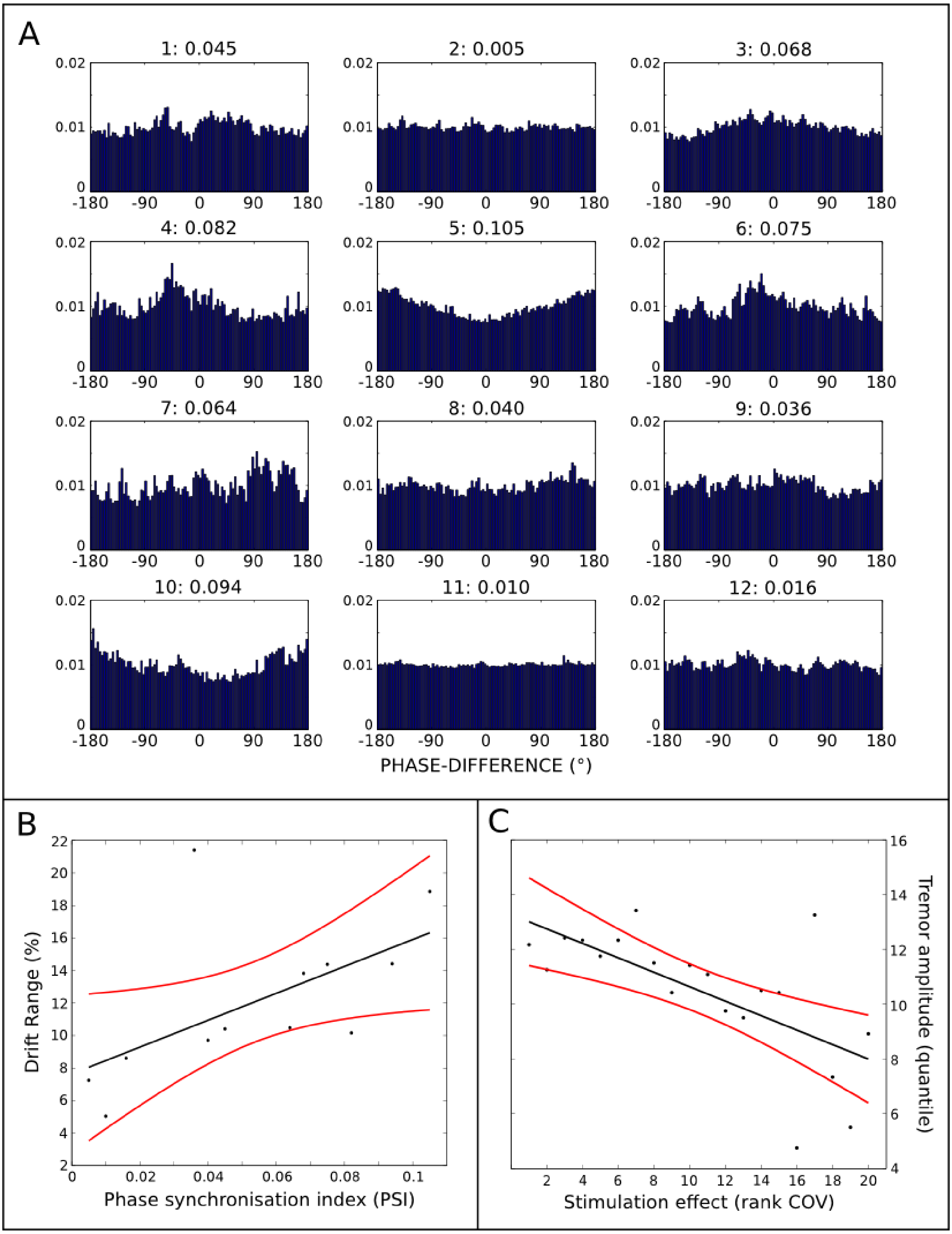


**Figure S2 (related to Figure.2).** (A) Phase-difference histograms between tremor and stimulation signals normalised as probability distributions. Labels correspond to patient number, followed by their derived phase-synchronisation index (PSI), where a PSI of 0 represents no entrainment and a 1

represents complete entrainment. Some patients display little to no phase-entrainment, whilst others show preponderance to certain phase-alignments. Note however that entrainment is modest and rarely approaches 0.1. Several patients show asymmetries in their phase-alignment histograms. (B) Regression analysis reveals that PSI is a reasonable predictor of stimulation efficacy (as quantified by the range of the drift profile), displaying a positive correlation ($R^2$=0.34, $p<0.05$). However, entrainment capacity did not significantly impact on the frequency-shift observed during tremor-frequency stimulation ($R^2$=0.25, p=NS; FDR corrected). (C) Group analysis relating the strength of stimulation effect with tremor amplitude. Tremor amplitude was stratified into 20 quantiles per patient, with a drift profile computed for each. Since this will necessarily result in a series of drift profiles with monotonically increasing means, we assess the normalised dispersion (coefficient-of-variation; COV) of the drift response. The COV was then rank ordered to permit cross-patient comparison. Quintiles were averaged for each rank COV, providing a group summary of how tremor amplitude impacts upon the range of the drift response. Finally, regression analysis revealed a significant negative relationship between tremor amplitude and response range, i.e. stimulation was most effective during low amplitude tremors ($R^2$=0.44, $p<0.01$, see Figure S4). This result suggests that weaker rhythms may be more amenable to intervention, leading us to postulate that the tremor network may be most effectively disrupted during tremor onset following stochastic periods of tremor suppression or as seen after movement. Thus we might anticipate bigger stimulation effects when tests involve more prolonged periods that include voluntary movement.

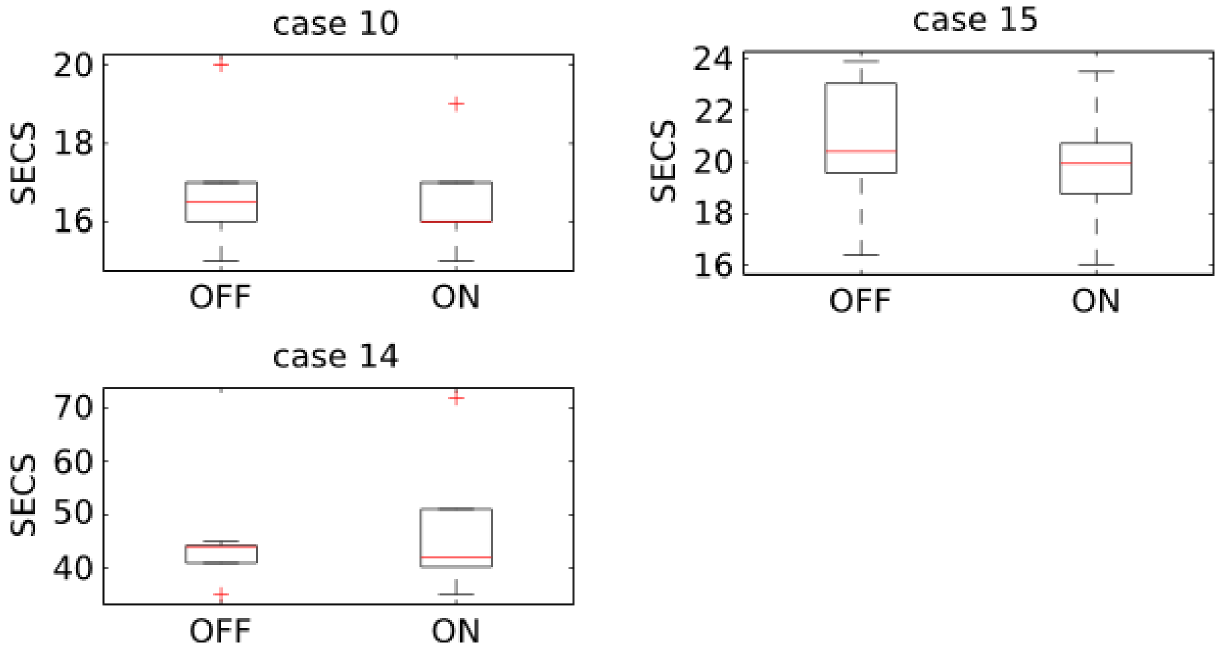


**Figure S3 (related to Figure.3).** As initial evidence that physiological processing remains unimpaired during phase-tracking at tremor-frequency stimulation, we present a sample of three patients who were recruited to perform an additional motor dexterity task. Patients were asked to sit at rest and allow their tremor to manifest. However, unlike in the main experiments, tremor was periodically interrupted by asking the patients to complete a 9-hole grooved pegboard test as quickly as possible with their tremulous hand. Patients were permitted to practise the pegboard test until comfortable before recording began. Each patient performed up to 20 repetitions of the test, with drift-derived suppressive phase-locked stimulation applied in half of all trials in a pseudo-randomised fashion. None of the patients showed a significant difference in pegboard performance between stimulation and sham conditions, as characterised by completion time (independent samples t-tests; case 10, t(18)=0.00, p=NS; case 14, t(8)=-0.72, p=NS; case 15, t(18)=0.91, p=NS).

| | Age (yrs) & Sex | Disease Duration (yrs) | Worse side | Levodopa daily equivalent dose (mg) [S1] |
|---|---|---|---|---|
| **1** | 77M | 5 | R | 652 |
| **2** | 68F | 7 | R | 720 |
| **3** | 82M | 4 | R | 150 |
| **4** | 74M | 4 | R | 300 |
| **5** | 62M | 3 | L | 45 |
| **6** | 74F | 2 | R | 325 |
| **7** | 71F | 3 | L | 105 |
| **8** | 78M | 2 | R | 0 |
| **9** | 70M | 10 | R | 200 |
| **10** | 76M | 3 | R | 300 |
| **11** | 69F | 2 | R | 300 |
| **12** | 77F | 3 | L | 300 |
| **13** | 62M | 3 | R | 150 |
| **14** | 60M | 3 | L | 0 |
| **15** | 46M | 3 | L | 0 |

**Table S1: Patient details**.

## Supplemental Experimental Procedures

Fourteen patients diagnosed with tremor-dominant Parkinson's disease were recruited (see Table S1). Patients were most affected by rest tremor in one upper limb, where the rest tremor score was 3-4 using item 20 of the motor unified Parkinson's disease rating scale. In some cases rest tremor invaded posture and slow manual activities such as finger taps, but higher frequency postural or action tremor was absent or grade 1 in this limb. Rest tremor in the other upper limb was grade 0-2, while high frequency postural or action tremor was absent or grade 1. Twelve patients participated in Experiment 1 and three of those patients also participated in Experiment 2, together with a further two cases. Recordings were performed following overnight withdrawal from their usual tremor medication.

Tremor was assessed by accelerometery (five using monoaxial EGAS-FS, Entran; ten using triaxial ACL300, Biometrics Ltd., UK). One axis of the accelerometer was oriented to traverse the wrist flexion/extension plane. All data were digitised at 1000 Hz and recorded by a Cambridge Electronic Design (CED; Cambridge, UK) 1401 analog-to-digital converter using CED Spike2 software. Data were downsampled (100 Hz) for offline analysis, conducted in Matlab (Mathworks, MA, USA).

The motor hotspot for the most pronounced tremor muscles in the worse affected upper limb was located by transcranial magnetic stimulation (TMS) over the contralateral motor region. The TACS electrode (5 x 7 cm) was centred over the hotspot with reference electrode (5 x 11 cm) secured to the ipsilateral (to tremor) shoulder with DC-centred stimulation delivered through a neuroConn (Ilmenau, Germany) DC Stimulator.

### Experiment 1: Phase drift

Following a brief recording to ascertain the patient's individual tremor frequency, TACS was applied in a randomised order over the following three conditions (1) at tremor frequency to the nearest Hz, (2) at double tremor frequency to the nearest Hz and (3) sham. Sham stimulation always began with a 30s period of stimulation at tremor frequency to replicate any sensations observed when the stimulator was switched-on. Stimulation was then rescinded for the remainder of the condition. Stimulation at double tremor frequency was applied in 10 of the 12 patients in Experiment 1. Each condition lasted for 10 minutes at a peak-to-peak stimulation current of 2 mA. There were no reported phosphenes, though several patients experienced mild skin irritation at the beginning of stimulation which always dissipated within 30s.

Acceleration from the wrist flexion/extension plane and TACS signals were zero-phase bandpass filtered (forward-backward filtering) off-line using a $3^{rd}$-order Butterworth filter (2Hz passband). Filtering was centred about the dominant tremor frequency in each case, accounting for any stimulation-induced change in tremor rhythm. Phase and amplitude information were extracted by Hilbert transform of the accelerometer. The phase of the TACS signal was analogously determined. In evaluating the results we must also contend with spontaneous variations in the intensity of parkinsonian tremor. Tremor amplitude information was therefore trend-corrected by applying 30s running average power normalisation, which focussed our drift analysis on local changes due to phase-alignment rather than undulating trends at very low frequency in the data. Phase information from both signals was quantised into 40 discrete bins (9° each).

To assess the influence of phasic stimulation on tremor amplitude, the mean amplitude of tremor was computed for all combinations of phase between TACS and tremor signals. These maps were smoothed (2D box kernel; 5 x 5 bins) and peak excitation and suppression amplitudes extracted. This permitted the efficacy of stimulation to be assessed even when stimulation and tremor frequencies were distinct (such as during first harmonic stimulation). Peak excitation and suppression amplitudes

were assessed between stimulation frequencies (tremor frequency versus harmonic stimulation) by repeated measures *t*-test, corrected for multiple comparisons through the FDR procedure (see [S2]).

Where the tremor and stimulation frequencies were similar (i.e. during tremor-frequency stimulation), the diagonal nature of the resulting maps reduced our analysis to an examination of phase-differences only, which was analogously smoothed (3 bins). The point-wise confidence intervals of Figure.1A are derived by a similar procedure applied to the sham condition, replacing the stimulation channel with white noise (which is subsequently filtered about the patient's tremor frequency). For convenience we describe these results in terms of the classical four quadrant model (see Figure.1), with quadrants I (0-90°), II (90-180°), III (180-270°) and IV (270-360°).

Orientation angles for the peak excitation and suppression response were extracted per patient and the mean resultant length (MRL) of these unit vectors computed [S3]. The MRL was assessed for statistical significance by non-parametric testing against an empirical null-distribution. The null-distribution was constructed by computing the MRL statistic over (cohort size) randomly oriented unit vectors, repeated 10,000 times. Significance was assessed at the level with FDR correction for multiple comparisons.

## Experiment 2: Phase tracking

Having validated the drift analysis as a suitable technique for determining preferential phase, we used the drift determined optimal phase for tremor suppression and delivered stimulation at this phase over 30s periods in a subset of five patients. Three of these had participated in Experiment 1 and agreed to further testing. A further two cases were recruited specifically for Experiment 2.

Tracking was accomplished by feeding the accelerometer signal into a CED Power1401 which operated as a real-time computer. In-house code (available on request) was developed (1401

conditioner code) to provide a sinusoidal output, phase-locked and offset from the reference tremor signal. No running normalisation was applied during tracking. Accelerometer phase was tracked by repeated (updating every 3 msecs) online evaluation using a Fast Fourier Transform (FFT) over the preceding 1 sec of data. Oscillatory phase at the tremor frequency (to the nearest Hertz) was identified and a phase-offset sinusoid output provided the phase-locked signal, with initial ramping in amplitude (a "soft-start"). This phase-locked sinusoid was fed into a TACS unit equipped with a bespoke "remote" function, permitting stimulation with an arbitrary waveform input. The attained phase relationship between TACS and tremor signals was monitored online, permitting fine-tuning adjustment to account for any phase bias as required. Together, this formed the basis of our closed-loop system. The mean amplitude of each 30 sec tracking segment was assessed (without power normalisation) and the dynamic range of the response presented as percentage change values relative to the grand mean of all 30s segments in that subject.

Following drift analysis we sought to validate our approach as a suitable technique for determining preferential phase. In one patient (case 10) from experiment 1 we stimulated (on a separate day) at 40 distinct phase angles for 30s each. This permitted reconstruction of the drift analysis with sustained stimulation and allowed us to assess the effect of sustained stimulation on tremor amplitude.

To confirm that our results related specifically to the phase of stimulation, we construct phase-difference histograms during sustained blocks of tremor suppression and excitation. This allows us to account for natural undulations in tremor amplitude as-well as any tracking instabilities that may occur. To begin, each of the 40 segments was categorised as excitatory, suppressive or unchanged relative to baseline. This was achieved by splitting each segment into 10 subdivisions (of 3 secs each), deriving the mean tremor amplitude per subdivision, then applying a single-sample t-test on each group relative to the mean tremor amplitude observed over the 10 min sham recording (repeated at the start of Experiment 2). Phase-difference histograms were constructed per segment

and averaged within each category. The preferential phase-alignment attained during periods of excitation and suppression relative to sham could then be assessed by considering the respective histograms. Excitatory and suppressive histograms can then be normalised to the no-change condition to identify regions of phase attraction (heightened incidence represented by positive values) and/or repulsion (reduced incidence represented by negative values) while the tremor is being driven or suppressed by stimulation.

## Supplemental References

[S1] Tomlinson CL, Stowe R, Patel S, Rick C, Gray R, Clarke CE. (2010) Systematic review of levodopa dose equivalency reporting in Parkinson's disease. Mov Disord. 25(15):2649-53.

[S2] Curran-Everett, D. (2000) Multiple comparisons: philosophies and illustrations. Am J Physiol Regul Integr Comp Physiol, 279: 1-8.

[S3] Lakatos, P., Chen, C. M., O'Connell, M. N., Mills, A. and Schroeder C. E. (2007) Neuronal Oscillations and Multisensory Interaction in Primary Auditory Cortex. Neuron, 52(2): 279-92.
